# A functional polymorphism in the *SPINK5 *gene is associated with asthma in a Chinese Han Population

**DOI:** 10.1186/1471-2350-10-59

**Published:** 2009-06-17

**Authors:** Qiji Liu, Yu Xia, Wenjing Zhang, Jisheng Li, Pin Wang, Huaichen Li, Chunhua Wei, Yaoqin Gong

**Affiliations:** 1 Department of Medical Genetics and Key Laboratory for Experimental Teratology of the Ministry of Education, Shandong University School of Medicine, Jinan, Shandong 250012, PR China; 2 Department of Respiratory Internal Medicine, Shandong Province Hospital, Jinan, 250001, PR China; 3 Weifang Asthma Hospital, Weifang, 264100, PR China

## Abstract

**Background:**

Mutation in *SPINK5 *causes Netherton syndrome, a rare recessive skin disease that is accompanied by severe atopic manifestations including atopic dermatitis, allergic rhinitis, asthma, high serum IgE and hypereosinophilia. Recently, single nucleotide polymorphism (SNP) of the *SPINK5 *was shown to be significantly associated with atopy, atopic dermatitis, asthma, and total serum IgE. In order to determine the role of the *SPINK5 *in the development of asthma, a case-control study including 669 asthma patients and 711 healthy controls in Han Chinese was conducted.

**Methods:**

Using PCR-RFLP assay, we genotyped one promoter SNP, -206G>A, and four nonsynonymous SNPs, 1103A>G (Asn368Ser), 1156G>A (Asp386Asn), 1258G>A (Glu420Lys), and 2475G>T (Glu825Asp). Also, we analyzed the functional significance of -206G>A using the luciferase reporter assay and electrophoresis mobility shift assay.

**Results:**

we found that the G allele at SNP -206G>A was associated with increased asthma susceptibility in our study population (p = 0.002, odds ratio 1.34, 95% confidence interval 1.11–1.60). There was no significant association between any of four nonsynonymous SNPs and asthma. The A allele at -206G>A has a significantly higher transcriptional activity than the G allele. Electrophoresis mobility shift assay also showed a significantly higher binding efficiency of nuclear protein to the A allele compared with the G allele.

**Conclusion:**

Our findings indicate that the -206G>A polymorphism in the *SPINK5 *is associated with asthma susceptibility in a Chinese Han population.

## Background

Asthma, a chronic inflammatory disease of the airway, is caused by inappropriate immune responses to environmental allergens. The individual variation in predisposition to asthma is genetically determined [1,2]. It is characterized prominently by an elevated production of immunoglobin E (IgE) and TH2 cytokines, a remarkable increase in recruitment of mast cells, eosinophils, and basophils to the airway epithelium as well as airway hyper-responsiveness (AHR) [3-5]. Pathogenesis of asthma is considered to be multifactorial, involving complex interactions between genetic and environmental factors [6]. Recently, considerable effort has been directed toward a better understanding of the genetic factors involved in the development of asthma and other allergic diseases. A number of asthma susceptibility genes have been identified through genome-wide screens and candidate gene studies [7,8]. Moreover, some genes that are responsible for monogenic disorders were found to contribute to manifestations of similar phenotype in complex diseases. For example, while mutations in *SPINK5*, causes Netherton syndrome, a rare autosomal recessive disorder, *SPINK5 *also plays a role in common atopic diseases [9,10].

The *SPINK5 *encodes the lymphoepithelial Kazal-type-related inhibitor (LEKTI), a 15-domain serine protease inhibitor, and is located on chromosome 5q31-32[11], a region repeatedly found to be linked to asthma [12-15]. Loss of function mutations in the *SPINK5 *gene cause Netherton syndrome, characterized by congenital ichthyosis with defective cornification, and severe atopic manifestations including atopic dermatitis, allergic rhinitis, asthma, markedly elevated serum IgE and hypereosinophilia [9]. Subsequently, Walley et al. identified 32 SNPs within the coding region of the *SPINK5 *gene, including six SNPs that result in amino acid changes in the encoded protein. They showed an association between the SNP G1258A (Glu420Lys) and atopy, atopic dermatitis, elevated serum IgE levels and asthma [10]. However, the results were not consistently replicated in other studies [16-20]. To test whether *SPINK5 *plays a role in the development of asthma in Han Chinese, a case-control study was conducted on 669 unrelated asthma patients and 711 healthy control subjects recruited in a Han Chinese population. Four nonsynonymous SNPs, 1103A>G (Asn368Ser), 1156G>A (Asp386Asn), 1258G>A (Glu420Lys), and 2475G>T (Glu825Asp), were genotyped, and no statistically significant difference in the genotype and allele distribution for any of four nonsynonymous SNPs was observed between the patients and control subjects. However, we found that the SNP -206G>A in promoter region was associated with asthma susceptibility. Furthermore, we analyzed the functional significance of -206G>A using luciferase reporter gene assay and electrophoretic mobility shift assay.

## Methods

### Subjects

Chinese subjects of exclusively Han nationality (n = 1380) were included in this case-control study. A total of 669 patients with clinically manifested asthma, 317 males and 352 females were recruited from Weifang Asthma Hospital and Shandong Provincial Hospital from 2004 to 2009. The extended medical history, including occurrence and duration of wheezing symptoms, was recorded for each patient. Unrelated, random-sampled controls, 349 males and 362 females had no symptoms of any atopic, lung and skin diseases and had no familial history of asthma/atopy. Clinical and demographic characteristics of the patients and controls are summarized in Table 1. Blood samples were obtained from all participants with informed consent. Genomic DNA was extracted from peripheral blood leukocytes by a standard salting-out method. The study protocol was approved by the ethics review committee for human studies at School of Medicine, Shandong University.

**Table 1:** Clinical and demographic characteristics of the patients and controls

Characteristic	Patients	Controls
No. of subjects	669	711
Age:mean (range)	41.27(14–73)	44.19(18–68)
Sex (male/Female)	317/352	349/362
FVC (%predicted)	66.9 ± 15.7	79.4 ± 18.4
FEV_1_(%predicted)	57.2 ± 16.6	83.8 ± 13.9
% changes of FEV_1 _by bronchodilator	26.8 ± 13.7	4.7 ± 3.6

FVC: forced vital capacity; FEV_1_: forced expiratory volume in 1s.

### SNP Genotyping

Five SNPs, one promoter SNP, -206G>A (transcription start site as +1), four nonsynonymous SNPs, 1103A>G (Asn368Ser), 1156G>A (Asp386Asn), 1258G>A (Glu420Lys), and 2475G>T (Glu825Asp), were genotyped by PCR-RFLP assay. DNA fragments containing polymorphic sites were amplified, and the primer sequences are shown in Table 2. PCR amplification was performed in a total volume of 25 μl solution containing 50 ng of genomic DNA, 200 μM of each dNTP (mixture of dATP, dTTP, dCTP, dGTP), 0.2 μM of each primer, 1.5 mM of MgCl2, 10 mM of Tris-HCL (pH 8.3) and 1 U of Taq DNA polymerase. Cycling conditions included an initial denaturation at 94°C for 5 minutes followed by 35 cycles at 94°C for 40 seconds, 55°C for 40 seconds, 72°C for 60 seconds, and a final extension at 72°C for 10 minutes. PCR products were digested with locus-specific restriction enzymes (Table 2). Digestion products were separated on 2% agarose gel and visualized by ethidium bromide staining.

**Table 2:** PCR-RFLP analysis of the SPINK5 gene SNPs

Polymorphic Site	Primers	PCR products	Restriction enzyme
-206 G>A	5'-TCTGCCTGCTCCAACTAA-3' 5'-CACTGACACTGTGGCTATCTT-3'	674 bp	Kpn I
1103A>G (Asn368Ser)	5'-TCTGCCAATGTAGATGTT-3' 5'-CTCTAATGACTCCCAAGC-3'	670 bp	Bts I
1156G>A (Asp386Asn)	As above	670 bp	Mbo I
1258G>A (Glu420Lys)	5'-TTTTAGCCAAGCAGAAGAAG-3' 5'-CAGTTTTAAGGAATGCACGT-3	177 bp	Hinf I
2475G>T(Glu825Asp)	5'-TGGGGAACTGAAGAGCA-3' 5'-GGGACATTATTTTGCCTATC-3'	688 bp	BstF5I

### Luciferase assay

Two luciferase reporter constructs, pGL3/-206G and pGL3/-206A, were generated by PCR combining with restriction digestion. The transcription start site of *SPINK5 *was according to the ensemble website . Briefly, a fragment of 487 bp in the *SPINK5 *promoter corresponding to -355 to +132 was amplified from two individuals, who are homozygous for -206G and -206A, using primers 5'-acgagctccccacttatgaagggagt-3' and 5'-ccgctcgagtggcaagaggcttagatt-3', which incorporate specific cleavage sequences for restriction endonucleases SacI and XhoI, respectively. The amplified PCR products were digested with SacI and XhoI, and the cleaved product was cloned into promoterless pGL3-basic plasmid (Promega). Plasmid constructs were verified by direct sequencing analysis. Plasmid DNA was prepared using plasmid mega kit (Omega) for further transfection.

Luciferase activity was measured using the Dual Luciferase Reporter Assay System (Promega). Briefly, COS-7 cells and HeLa cells were cultured in DMEM (Invitrogen) supplemented with 10% fetal bovine serum, 100 IU/ml penicillin, and 100 ug/ml streptomycin at 37°C in an atmosphere of 5% CO_2_. Cells, 5 × 10^4^/well, were inoculated in a 24-well plate and cultured for 24 h before transfection. The subconfluent cells were cotransfected with 1 μg of each construct (pGL3/Basic, pGL3/-206G and pGL3/-206A) and 0.04 μg of pRL-TK vector, an internal control for transfection efficiency, using Lipofectamine 2000 transfection reagent (Invitrogen). Thirty-six hours after transfection, cells were harvested and lysed with passive lysis buffer (Promega) according to the manufacturer's instructions. Luciferase activity was measured using a Microplate luminometer (3700) and normalized using the activity of the Renilla luciferase. The relative luciferase activity of the *SPINK5 *reporter construct was represented as the ratio of the firefly luciferase activity to that of Renilla. Each experiment was repeated three times, and each sample was studied in triplicate.

### Promoter database analysis

Allele specific transcription factor binding sites were identified using MatInspector software 

### Electrophoretic Mobility Shift Assay (EMSA)

EMSA was performed as described previously [21]. Briefly, nuclear proteins were extracted from HeLa cells using the NE-PER nuclear and cytoplasmic extraction reagents according to the manufacturer's protocol (Pierce, Rockford, IL). The sense-strand sequences of -216A probe (containing GATA binding site) and -216G probe (without GATA binding site, as a competitor) were 5'-GTTCTGGGGGAGATACCATGAAAGA-3' and 5'-GTTCTGGGGGAGGTACCATGAAAGA-3', respectively. The oligonucleotides of -216A probe and their complementary strand were annealed and end-labeled with digoxin using T4 polynucleotide kinase. DIG-labeled probes were incubated with or without 5 μg HeLa nuclear extracts for 30 min at room temperature and separated on a 6% non-denaturing polyacrylamide gel with 0.5× TBE running buffer. DNA-protein complexes were electroblotted onto nylon membrane and the band shift was visualized according to the user's manual for DIG Gel Shift Kit. For the competition assay, nuclear extracts were incubated with 25 or 50-fold amount of unlabeled probes for 15 min prior to adding labeled probe.

### Statistical analysis

Allele carrier frequency was defined as the percentage of the individuals carrying the allele among the total of the individuals. Each SNP was evaluated for Hardy-Weinberg equilibrium using a χ^2 ^goodness of fit test. The asthma patients and healthy controls were compared using the case-control association analysis. Fisher's exact test was used to compare the discrete variables between asthma patients and healthy controls. The allele and genotype frequencies, odds ratios (ORs) with 95% confidence intervals (95%CI), and significance values were analyzed using SPSS software for Windows (version 13; SPSS Inc, Chicago, IL). Pairwise linkage disequilibrium (LD) was measured using the online SHEsis software . The luciferase reporter assay data were evaluated using a non-parametric Mann-Whitney test (two-tailed). Statistical analysis was performed with the SPSS 13.0 software. Power calculation was performed using the computer program CaTS power calculator to evaluate the type II error [22]. A p-value less than 0.05 were considered statistically significant.

## Results

### Association of *SPINK5 *SNPs and haplotypes with asthma

Five SNPs genotyped in this study were chosen based on their putative function, four of which were nonsynonymous substitutions, one from the promoter region. Genotype and allele frequencies of each SNP are shown in Table 3. The distributions of five SNPs were in agreement with Hardy-Weinberg equilibrium (P > 0.05) in both asthma patients and healthy controls. To test the association between each locus and asthma, we compared differences in allele frequency and genotype distribution of each polymorphism between case and control subjects. We found that the G allele of SNP -206 G>A was significantly more common among patients with asthma than among the controls (P = 0.002, OR 1.34; 95% CI: 1.11–1.60). This elevated risk associated with G allele remains significant even after adjusted by Bonferroni correction (adjusted P = 0.01). This result suggested that SNP -206G>A in the *SPINK5 *promoter region was significantly associated with asthma in our Chinese cohort. On the other hand, no statistically significant associations between four nonsynonymous *SPINK5 *SNPs and asthma were observed. With our sample size and with the alpha level set at 0.05, there is at least 80% power for detection of OR ≥1.3 for each of these four SNPs.

**Table 3:** Genotype and allele frequencies of the *SPINK5 *SNPs in patients and controls

SNPs	Genotype/allele	Controls(%)	Patients (%)	Odds ratio (95% CI)	*P*	P*
-206 G>A	AA	48 (6.75)	31 (4.63)	1.00		
	AG	243(34.18)	192 (28.70)	1.22(0.75–2.00)		
	GG	420(59.07)	446(66.67)	1.64(1.03–2.63)	0.01	0.05
	A	339 (23.84)	254(18.98)	1.00		
	G	1083(76.16)	1084(81.02)	1.34(1.11–1.60)	0.002	0.01
1103A>G	AA	74(18.05)	92(21.80)	1.00		
	AG	201(49.02)	192(45.50)	0.77(0.53–1.11)		
	GG	135(32.93)	138(32.70)	0.82(0.56–1.21)	0.365	
	A	349(42.56)	376(44.55)	1.00		
	G	471(57.44)	468(55.45)	0.92(0.76–1.12)	0.429	
1156G>A	GG	110(26.83)	111(26.30)	1.00		
	GA	201(49.02)	192(45.50)	0.95(0.68–1.32)		
	AA	99(24.15)	119(28.20)	1.19(0.82–1.73)	0.392	
	G	421(51.34)	414(49.05)	1.00		
	A	399(48.64)	430(50.95)	1.10(0.90–1.33)	0.352	
1258G>A	GG	174(42.44)	169(40.05)	1.00		
	GA	199(48.54)	201(47.63)	1.04(0.78–1.39)		
	AA	37(9.02)	52(12.32)	1.45(0.90–2.34)	0.295	
	G	547(66.71)	539(63.86)	1.00		
	A	273(33.29)	305(36.14)	1.13(0.93–1.39)	0.236	
2475G>T	GG	169(40.05)	191(46.59)	1.00		
	GT	201(47.63)	169(41.22)	0.97(0.74–1.32)		
	TT	52(12.32)	50(12.20)	1.07(0.69–1.65)	0.932	
	G	539(63.86)	551(67.20)	1.00		
	T	305(36.14)	269(32.80)	1.02(0.83–1.25)	0.835	

P* indicates adjusting after Bonferroni correction

To better understand the relationship between the five SNPs in *SPINK5*, linkage disequilibrium analysis was assessed. There is significant linkage disequilibrium between the SNPs (Table 4). We further analyzed the haplotype structure, and identified seven common haplotypes (Table 5). The frequency of the A-G-A-G-G haplotype (haplotype 3) in the control group was 16.8% and that in affected individuals was 11.7%. The difference was statistically significant (P = 0.043).

**Table 4:** Linkage disequilibrium coefficient (| D'|) among each polymorphism locus in SPINK5 gene*

Locus	1103A>G	1156G>A	1258G>A	2475G>T
-206G>A	0.628	0.769	0.616	0.680
1103A>G	-	0.929	0.854	0.855
1156G>A	-	-	0.821	0.896
1258G>A				0.777

*The pairwise linkage disequilibrium analysis was conducted by online software SHEsis.

**Table 5:** The haplotype frequencies of the five polymorphisms in asthma patients and healthy controls.

						Frequency		
Haplotypes	-206	1103	1156	1258	2475	Asthma	control	P*
Hap1	G	A	G	A	G	0.283	0.265	0.583
Hap2	G	G	A	G	T	0.275	0.260	0.691
Hap3	A	G	A	G	G	0.117	0.168	0.043
Hap4	G	A	G	G	G	0.112	0.109	0.896
Hap5	G	G	G	G	G	0.070	0.083	0.497
Hap6	G	G	A	G	G	0.031	0.021	0.370
Hap7	A	G	A	G	T	0.031	0.018	0.260

*The P value was calculated with the haplotype frequencies greater than 0.03 by online software-SHEsis.

### -206G>A polymorphism is associated with differential transcription activity

**Figure 1:**
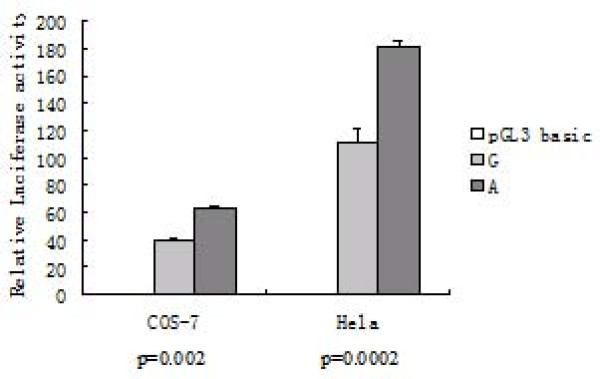
**Effect of the -206 G>A on the transcription activity of the *SPINK5 *promoter**. COS-7 cells or Hela cells were transiently cotransfected with pGL3/-206G or pGL3/-206A and pRL-TK vector. The relative luciferase activity of the *SPINK5 *reporter constructs is represented as the ratio of the firefly luciferase activity to that of Renilla. Each experiment was conducted in triplicate for each sample, and the results are expressed as mean ± SD for three independent experiments. P value was determined by the Student's t test.

The functional significance of -206G>A polymorphism in transcription was explored using MatInspector software. The G to A substitution at position -206 is predicted to generate a GATA-3 transcription factor binding site (5'-GATA-3'). To determine the effect of -206G>A polymorphism on promoter activity, allele-specific constructs, pGL3/-206G, and pGL3/-206A, were generated, and transiently expressed in COS-7 cells and HeLa cells. Luciferase activity in cell extracts was analyzed 36 hours after transfection and was standardized against internal control Renilla activity. Results indicated that the -206G construct showed a significant decrease in luciferase reporter activity compared with the -206A construct (*p *= 0.002 and *p *= 0.0002 for COS7 and HeLa cells, respectively, Fig. 1). These results suggested that the -206G allele might render a decreased transcriptional activity of the *SPINK5 in vivo*.

### -206G>A polymorphism is associated with differential binding by nuclear proteins

**Figure 2:**
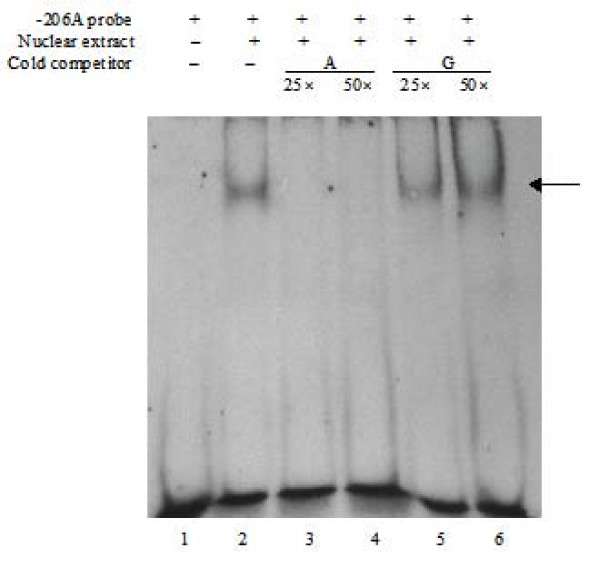
**EMSA showing the physical binding of nuclear extract to GATA binding site**. The arrow indicates DNA-protein complex formed when transcription factor binds to the target site. The retarded mobility of the oligonucleotide was observed in the presence of nuclear extract, lane 2. The formation of DNA-protein complex was competitively inhibited by 25- and 50-fold amount of unlabeled -206A probe, lanes 3 and 4, but not by the same amount of unlabeled -206G probe.

To further determine the requirement of the GATA binding site for the transcription regulation of *SPINK5*, EMSA was conducted to test the binding efficiency of nuclear proteins from the HeLa cells to the -216A probe. As shown in Figure 2, a retarded band (a DNA-protein complex) was observed in the presence of nuclear extracts (lane 2). The competition assay with the unlabelled oligonucleotides demonstrated that the DNA-protein complex was significantly competed by 25- and 50-fold amount of unlabeled -206A probe (lanes 3 and 4), but not by the same amount of -216G probes (Lanes 5 and 6). These results indicate that the -216A probe containing the GATA binding site specifically binds with endogenous GATA transcription factor. Creation of the GATA binding site by -216A allele probably contributes to an enhanced *SPINK5 *transcription.

## Discussion

Because Netherton syndrome exhibits allergic phenotype, it is reasonable to speculate that *SPINK5*, which is mutated in Netherton syndrome, may act as a candidate gene for asthma and other allergic diseases [9]. An extensive search for single nucleotide polymorphisms in the *SPINK5 *led to the identification of a number of SNPs, including six nonsynonymous SNPs in coding region that might perturb its immune function. Subsequent genotyping of three nonsynonymous SNPs, A1103G, G1156A and G1258A in two independent panels of British families showed a significant association between SNP G1258A (Glu420Lys) and atopy, atopic dermatitis, elevated serum IgE levels and asthma [10]. This association was confirmed in a large German population and two Japanese populations [16-18]. Kato et al analyzed eight SNPs in exon 13 and 14 of the *SPINK5 *including G1258A (Glu420Lys), and found a positive association of seven SNPs with atopic dermatitis in a Japanese study sample using a case-control study design [16]. Nishio et al surveyed five of six previously reported nonsynonymous *SPINK5 *SNPs in Japanese atopic families identified through asthmatic children or subjects with atopic dermatitis and found that *SPINK5 *was associated with development of atopic dermatitis but not asthma [17]. Kabesch M et al. analyzed G1258A (Glu420Lys) in a German population of school children, and found its association with asthma as well as a concomitant occurrence of asthma and atopic dermatitis [18]. However, two subsequent studies failed to replicate the original SPINK5 findings for allergic diseases [19,20]. Folster-Holst et al genotyped four nonsynonymous SNPs (Asp106Asn, Asn368Ser, Asp386Asn, and Glu420Lys), and detected no association between *SPINK5 *and atopic dermatitis in populations of Northern German origin [20]. Jongepier H et al. failed to detect any association between *SPINK5 *and asthma, atopic phenotypes and atopic dermatitis in a Dutch population [19]. These discordant findings probably reflect different genetic and environmental backgrounds in various populations. To determine whether nonsynonymous SNPs of the *SPINK5 *are involved in the pathogenesis of asthma in the Chinese Han population, we performed a case-control study by genotyping four nonsynonymous SNPs in the *SPINK5*. We did not detect any significant association between these nonsynonymous SNPs and asthma in our Chinese samples. With our sample size, we expected a power of at least 80% in detecting an effect of OR ≥1.3 for each of these SNPs. Therefore, our failure to detect an association for these 4 SNPs was not due to the sample size. These results suggest that the polymorphisms in the coding region of the *SPINK5 *are unlikely to contribute to asthma risk in the Chinese Han population. However, because our patients were ascertained for asthma, we could not exclude a role of the coding SNPs of the *SPINK5 *in atopic dermatitis in our population.

The variations in the regulatory sequences of genes may determine risks to common diseases by causing different levels of expression. Therefore, the identification and functional evaluation of polymorphisms in promoter region are of great value in understanding the genetic susceptibility to asthma. In order to determine the role of the *SPINK5 *promoter polymorphism in the pathogenesis of asthma, we genotyped a promoter polymorphism, -206G>A, in 422 asthma patients and 410 controls, and found a marginal association. The frequency of allele G was significantly higher in asthmatic patients than that in controls (p = 0.022). To confirm the association, additional 267 asthma patients and 301 controls newly recruited from the same hospital were genotyped, and the -206G>A polymorphism remained significantly associated with asthma (P = 0.001), even after Bonferroni correction(adjusted P = 0.01). To our knowledge, this is the first report of an association of -206G>A polymorphism with asthma. We further examined the potential functional role of this promoter polymorphism, and found that the G to A substitution at -206 generated a GATA-3 transcription factor binding site.

Major transcription factors controlling Th1 and Th2 development, such as T-box transcription factor and GATA3, are possibly involved in asthma and atopic diseases. GATA-3, a transcription factor specifically expressed in T helper 2 (Th2) cells, plays a critical role in the differentiation of Th2 cells from uncommitted CD4+ lymphocytes. In addition, GATA-3 is essential for the expression of the cytokines IL-4, IL-5 and IL-13 that mediate allergic inflammation [23]. Our luciferase reporter assay confirmed that SNP -206G>A is associated with the transcriptional activity of *SPINK5*. The G allele was associated with decreased transcriptional activity of the *SPINK5*. The mechanism by which the -206G>A SNP affects *SPINK5 *expression may be explained by the potential differential transcription factor binding of GATA binding factor, since -206G>A is located at the core sequence of GATA binding factor binding site. Electrophoretic mobility shift assay confirmed that the A to G substitution at -206 significantly reduced the binding efficiency of nuclear proteins to this element. Our data suggest that loss of GATA transcription factor regulation with the -206G may decrease the *SPINK5 *expression and thereby potentially perturb the immunosuppressive function of LEKTI.

## Conclusion

In summary, although previous studies indicated that coding SNPs in the *SPINK5 *might be associated with asthma, we were unable to detect an association between the polymorphisms in the coding region of the *SPINK5 *and asthma risk in the Chinese Han population. However, we detected a significant association between -206G>A polymorphism in *SPINK5 *promoter and asthma. A single base substitution at -206 generated a functional promoter element and altered the transcriptional activity of the *SPINK5*.

## Competing interests

The authors declare that they have no competing interests.

## Authors' contributions

QL performed the PCR-RFLP and EMSA experiments. YX performed luciferase assay. WZ contributed plasmid construction. JL and PW participated in the DNA sample preparation. HLand CW collected the patient. QL and YG wrote the paper. All authors read and approved the final manuscript.

## Pre-publication history

The pre-publication history for this paper can be accessed here:


